# Health-seeking behaviour for schistosomiasis: a systematic review of qualitative and quantitative literature

**DOI:** 10.11604/pamj.2013.16.130.3078

**Published:** 2013-12-05

**Authors:** Thomas Cronin, James Sheppard, Gilles de Wildt

**Affiliations:** 1 College of Medical and Dental Sciences, University of Birmingham, UK; 2 School of Health and Population Sciences, Primary Care Clinical Sciences, University of Birmingham, UK

**Keywords:** Health-seeking behaviour, schistosomiasis, community, cobtrol, case finding

## Abstract

Schistosomiasis is a chronic and debilitating parasitic disease acquired through contact with infested freshwater. An essential component of its control is passive case finding, which, in order to be effective, requires a detailed understanding of health-seeking behaviour. This study aimed to systematically review evidence on health-seeking behaviour for schistosomiasis, in order to determine factors influencing use or non-use of modern health services for the infection. Quantitative, qualitative and mixed method studies reporting on factors related to seeking treatment from modern health services for schistosomiasis were obtained, combining electronic and hand searching. Data extraction and quality assessment of the included articles were performed, with all studies qualitatively analysed using thematic synthesis. A total of 19 studies were included in the review. Six themes were identified from the analysis: biomedical knowledge on schistosomiasis, perceptions of modern treatment and health services, financial considerations of treatment, perceptions on the symptoms, stigma of the infection, and physical location and community. These findings were consistent across studies of different design, setting and quality. Many of the themes identified echo existing literature on health-seeking behaviour. The synthesis also highlighted the role of stigma, and aspects of the physical location and community that may affect treatment-seeking for schistosomiasis. Health education programmes that intend to improve the utilisation of modern health services for the infection need to acknowledge the multiple determinants influencing their use. Future research should move beyond describing health-seeking behaviour to identifying the factors that underlay such behaviour.

## Introduction

Schistosomiasis is a chronic and debilitating parasitic disease acquired through contact with infested freshwater [1]. It is especially common in sub-Saharan Africa where over 90% of the global burden is concentrated [2]. An estimated 207 million people are infected with the disease, and more than 800 million live in transmission zones [3].

The effects of the condition are varied. Haematuria and dysuria constitute the main early symptoms of urinary schistosomiasis (caused by *Schistosoma haematobium*), whilst its long-term effects include renal failure and bladder carcinoma. Intestinal schistosomiasis (caused by *S. mansoni*, *S. japonicum*, *S. intercalatum*, and *S. mekongi*) gives rise to bloody diarrhoea and abdominal pain, with later-stage complications resulting from portal hypertension. Furthermore, growth stunting and anaemia can be outcomes of the infection [4].

Death rate from schistosomiasis infection is relatively low. Quality of life is significantly affected, with estimates of the disability-adjusted life years (DALYs) lost annually as high as 70 million [4]. This figure is comparable with the DALYs lost through HIV/AIDS and exceeds that of malaria or tuberculosis [5]. Despite this, schistosomiasis remains underdiagnosed and undertreated, leading to its inclusion within the World Health Organisation's (WHO) Neglected Tropical Diseases list [6].

Currently, the main principle of schistosomiasis control, as advocated by the WHO, is the concept of morbidity control and its implementation within the primary health care system [7]. This involves treating the infection in order to lower its intensity, in contrast to transmission control, which aims at reducing its prevalence [8].

An essential component of morbidity control is the provision of adequate clinical care for patients presenting at modern health services with early signs and symptoms of the infection (passive case finding). In areas without any form of organised schistosomiasis control, passive case finding is the only option for control [9]. Moreover, with pathology being strongly related to intensity and duration of infection, early treatment of schistosomiasis infection can prevent most of the severe morbidity later [10]. Thus, passive case finding represents an important aspect of schistosomiasis control, that is particularly feasible given the relative inexpensive price of the anti-helminth medication, praziquantel ($0.07 USD per tablet, excluding distribution costs) required as a single dose [8].

For passive case finding to be an effective control measure, a detailed understanding of health-seeking behaviour for schistosomiasis is required [11]. Gaining this knowledge of health-seeking behaviour is prerequisite to developing health care systems and health education programmes sensitive to the local dynamics of their communities [12–14]. By achieving this, benefits could include reducing the delay to diagnosis to improving health promotion strategies [12].

Reviews of the literature on schistosomiasis have revealed the increasingly important role of social sciences in the control of schistosomiasis [8, 15, 16]. These reviews, however, have not focussed on health-seeking behaviour, and have not employed a systematic approach.

**Review objective**: The purpose of this paper is to review the available literature on health-seeking behaviour in relation to schistosomiasis. Specifically, it aims to answer the following research question: What are the factors that determine use or non-use of modern health services for schistosomiasis infection? In this review, modern health services refer to health centres, clinics, health facilities and hospitals.

## Methods

### Search strategy

A MEDLINE search strategy was developed and then adapted for use in PubMed, Web of Science, CINAHL (the Cumulative Index to Nursing and Allied Health Literature) and AJOL (African Journals OnLine). Scoping searches were carried out beforehand to refine the search terms and ensure that relevant studies were obtained. The search terms and strategy used are described in Table 1. Further hand searches were performed using the bibliographies of identified publications, and articles that had cited these publications were screened to further identify appropriate studies. All databases were searched in March 2012.

**Table 1:** Search terms used and databases accessed

	PubMed	Web of Science	CINAHL	AJOL
	schisto* OR bilharz* OR helminth*	schisto* OR bilharz* OR helminth*	schisto* OR bilharz* OR helminth*	schisto* OR bilharz* OR helminth*
**AND**	help OR utilization OR behaviour OR seek* OR practice* OR perception* OR attitude* OR belief*	help OR utilization OR behaviour OR seek* OR practice* OR perception* OR attitude* OR belief*	help OR utilization OR behaviour OR seek* OR practice* OR perception* OR attitude* OR belief*	
**Retrieved**	1143	1752	36	588

Citations and abstracts from the searches of PubMed, Web of Science, and CINAHL were exported to Endnote X4 (Thomson Reuters), with duplicates identified and removed. Retrieved articles from AJOL could not be entered within the reference management software, and were reviewed separately. All searches were carried out by TC.

### Study selection: inclusion and exclusion criteria

Only English language articles and human studies were included and no date limits were applied. We considered all qualitative, quantitative, and mixed method studies that reported on factors related to seeking treatment from modern health services for schistosomiasis. Combining qualitative and quantitative data in a review may limit bias, improve reliability, and enhance accuracy of recommendations [17]. Other reviews and papers that did not report primary data were excluded from the analysis. Decisions on eligibility were made by the author TC.

### Data extraction and quality assessment

A standard data extraction form was used that covered both qualitative and quantitative studies. The data extraction form was developed using the Centre for Reviews and Dissemination guidance template [18]. It recorded basic information first (authors, date, title of paper and journal details), and then detailed information from the study (study design, study location, aims of the study, study population, and study findings related to the research question).

There is much debate surrounding whether quality criteria can be applied to systematic reviews of qualitative literature [19, 20], with some researchers considering the application of such criteria to be excessively reductionist [21]. Therefore, it was decided not to exclude any relevant studies on the basis of quality assessment. Instead, the aim of the quality assessment was to explore whether quality of the study affected the results.

The quality of included articles was assessed using a standardised grading scheme that could be used for both qualitative and quantitative literature [22]. Ten areas (title and abstract; introduction and aims; method and data; sampling; data analysis; ethics; bias; results; transferability or generalisability; and implications and usefulness) were awarded one of four grades: very poor (one point), poor (two points), fair (three points) and good (four points), providing a maximum score of 40.

### Data synthesis

The methods for systematically analysing both qualitative and quantitative data are still evolving [19, 23, 24]. The method applied for this study is similar to the thematic synthesis as described by Thomas and Harden [25]. Within this approach, all the relevant data were qualitatively analysed together, with no distinction being made according to the type of data. The analysis followed three stages, as recommend by Thomas and Harden [25], with the key difference being that ‘line by line’ coding was completed on paper rather than using software. In stages one and two, the extracted data was coded and ‘descriptive themes’ were developed, while in stage three, ‘analytical themes’ were formed. This process was carried out by TC and resulting themes, therefore, represent the author's own interpretation of the data.

### Current status of knowledge

**Figure 1:**
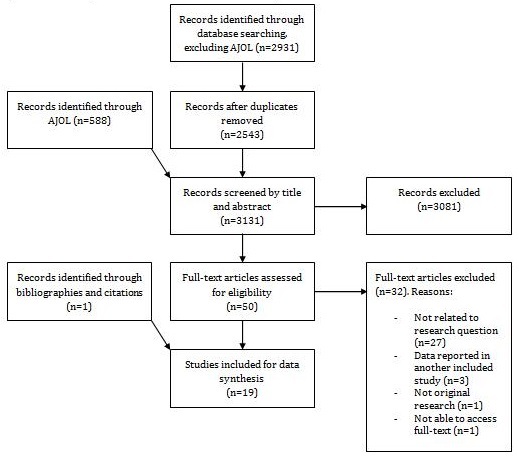
Summary of the article inclusion and exclusion process

A total of 3,131 studies were screened by title and abstract (Figure 1). After exclusion of 3,081 articles, the remaining 50 were screened by full-text. A total of 32 articles were excluded because they did not include primary data, were not related to the study question or were not original research papers. A total of 19 articles (the remaining 18 studies plus one additional article identified through hand searches of included studies reference lists) were entered into the analysis.

The included studies were heterogeneous in their design, setting, and quality (**Annex 1**). There were seven qualitative studies, six quantitative studies, and six mixed methods studies. The included research was conducted in Brazil (3 studies), Cameroon (2), Egypt (1), Ghana (4), Cote d'Ivoire (1), Kenya (1), Nigeria (3), Tanzania (3), and Uganda (1). Quality scores of the studies ranged from 22 to 37 (median score = 32, interquartile range = 9).

Analysis of the articles gave rise to 6 themes; 1) Biomedical knowledge on schistosomiasis; 2) Perceptions of modern treatments and health services for schistosomiasis; 3) Financial considerations of treatment for schistosomiasis; 4) Perceptions on the symptoms of schistosomiasis; 5) Stigma of schistosomiasis infection; and 6) Aspects of the local environment and the community (Table 2).

**Table 2:** Included studies that reported or discussed each identified theme (represented by X)

Reference	Biomedical knowledge on schistosomiasis	Perceptions of modern treatments and health services for schistosomiasis	Financial considerations of treatment for schistosomiasis	Perceptions on the symptoms of schistosomiasis	Stigma of schistosomiasis infection	Aspects of the local environment and community
Kloos *et al*. 1987 [39]		X	X	X		X
el Katsha & Watts 1997[26	X	X				X
Hewlett & Cline 1997 [35]		X	X	X		
Gazzinelli *et al*. 1998 [37]		X		X		X
Aryeetey *et al*. 1999 [27]	X	X		X	X	X
Uchoa *et al*. 2000 [41]		X				
Ahlberg *et al*. 2003 [33]		X	X		X	X
Akinwale *et al*. 2004 [40]		X				
Danso-Appiah *et al*. 2004^36^		X	X	X		
Mwanga *et al*. 2004 [30]	X	X		X	X	X
Takougang *et al*. 2004 [43]	X				X	
Mwanga, 2005 [31]	X					
Adeneye *et al*. 2007 [38]		X				
Anguzu *et al*. 2007 [28]	X	X				
Acka *et al*. 2010 [29]	X			X		
Danso-Appiah *et al*. 2010^42^			X	X		
Onyeneho *et al*. 2010 [34]		X				
Reis *et al*. 2010 [44]						X
Yirenya-Tawiah *et al*. 2011^32^	X	X	X			X

### Biomedical knowledge on schistosomiasis

Four studies related lack of knowledge on the infection's signs and symptoms as a factor for not seeking treatment [26–29]. Two studies identified that beliefs about supernatural causes of schistosomiasis resulted in traditional healers being consulted, in favour of modern treatment [30, 31]. In Ghana, one study found that subjects with higher educational attainments more often visited health services than those with lower educational attainments [32].

### Perceptions of modern treatments and health services for schistosomiasis

Three studies found that treatment was regarded as futile [27, 33, 34]. Some participants perceived the infection as untreatable due to its ever-presence in the area [27]. This was similar across settings, including Tanzania, where respondents felt unless infested waters were treated, they would continue to become infected [33].

Perceived inadequacy of health services was reported as deterring people from obtaining treatment (three studies) [26, 35, 36]. Similarly, unavailability of medications from health services was also recorded as a factor for not seeking treatment (four studies) [30, 35, 37, 38].

Preferences to self-treat the infection using herbal remedies or medications obtained from local markets resulted in health services not being consulted (four studies) [36, 38–40]. Participants from three of these studies attributed lower cost and higher accessibility, relative to health services, for self-treating [38–40].

In comparison, reasons for seeking modern treatment related to its perceived effectiveness (eight studies) [30, 33, 34, 37–41]. Failure of other treatments, such as herbal remedies, also resulted in modern treatment being utilised (two studies) [38, 39].

### Financial considerations of treatment for schistosomiasis

Unaffordability of modern treatment and health services was a commonly cited factor for not seeking treatment for schistosomiasis (six studies) [33, 35, 36, 39, 40, 42]. Danso-Appiah et al. [36] and Yirenya-Tawiah et al. [32] reported that 47% (n = 214) and 74% (n = 654) of the reasons for not seeking modern treatment for schistosomiasis infection related to lack of finances, respectively. This was consistent with the finding in another study that those in higher socio-economic groups were more likely to seek treatment for the infection. This study also found that someone else paying for health services positively influenced whether modern treatment was sought [42]. Interestingly, a study in Cameroon observed affordability of treatment varied according to the season within the year. Participants commented they would more likely obtain treatment after the cotton harvest, when they usually received a lump sum payment for their crop [35].

### Perceptions on the symptoms of schistosomiasis

Seven studies reported perceived lack of severity for schistosomiasis symptoms negatively influenced whether action was taken [27, 30, 35–37, 39, 42]. Danso-Appiah et al. [36] recorded that 44% (n = 203) of the reasons for not seeking modern treatment related to perceived lack of seriousness of the symptoms. Accordingly, when symptoms were perceived as serious, health services were utilised (three studies) [29, 37, 42]. Where participants felt symptoms went away on their own, treatment was unlikely to be sought (five studies) [30, 31, 34, 35, 43].

### Stigma of schistosomiasis infection

Some studies indicated there was reluctance to seek treatment for schistosomiasis due to its being perceived as an embarrassing and shameful condition (three studies) [30, 33, 43]. Research in Tanzania suggested that some participants believed urinary schistosomiasis to be sexually transmitted and as a result, may delay seeking treatment [33]. In Ghana, some children with the condition were unwilling to inform their parents for fear of punishment [27].

### Aspects of the local environment and the community

Three studies reported that difficulty in accessing health services resulted in some participants not seeking treatment for schistosomiasis [32, 37, 44]. A further three studies highlighted that dependence on external control measures in the area, for treatment of the disease, may result in health services not being used [26, 27, 44]. For example, in Egypt, parents failed to obtain treatment for their children due to reliance on the school-based testing and treating system [26].

In Kenya, social pressure from neighbours and relatives was commonly placed on mothers to take their children, with severe symptoms, to the health services [39]. In some instances, it was reported that traditional healers referred patients with schistosomiasis-symptoms onto modern health services (three studies) [30, 37, 39].

### Main findings of the review

This review has identified six themes that influence the use or non-use of treatment provided by modern health services for schistosomiasis infection. On the whole, themes were congruent between studies, irrespective of quality. This also applied to location and methodology, with the same themes emerging from studies of different design and setting. Such congruency suggests the synthesised findings of this review are likely to be reliable.

Many of the identified themes echo the existing literature on health-seeking behaviour, i.e. biomedical knowledge, perceptions of modern treatment and health services, financial considerations, and perceptions of symptoms [45, 46]. Furthermore, such themes have been noted in reviews of social science literature in relation to malaria [47, 48].

Our findings draw attention to the potential stigma attached to this infection, which could result in treatment not being obtained [27, 30, 33, 43]. Stigma has rarely been discussed in social science literature surrounding schistosomiasis, and has previously been identified as an issue rarely researched [8].

This review has also highlighted how aspects of the local environment and community influence the decision making process to obtain treatment. For example, it is understandable that an external schistosomiasis control programme in the area that actively treats the infection may result in local health services not being used [26, 27, 44]. However, the long-term effects on health-seeking behaviour following the end of such strategies are unclear.

Lastly, this study has demonstrated the complexity of health systems that often exist in communities where schistosomiasis is endemic, with sources of treatment ranging from self-care to traditional and modern medicine. In some cases, there appears to be a synergy between traditional healers and modern health services [30, 37, 39], providing further evidence for their potential role within a health system [49].

### Limitations of this review

The review employed a comprehensive search strategy, combining electronic and hand searching. However, the exclusion of non-English language articles could have limited the number of relevant articles identified. A previous review on social science literature and schistosomiasis included relevant studies in Portuguese and French. Some of these foreign language studies appear likely to be applicable within this review [8]. In addition, the full-text of one potentially relevant paper could not be accessed, with the abstract indicating potentially useful information could have been obtained.

The absence of a second reviewer involved in the data extraction, quality assessment and data synthesis processes, owing to time restrictions, represents a further weakness of the study. In addition, the analysis process employed within this review to collate qualitative and quantitative data is still developing [20, 24]. In any case, it has been argued that synthesising qualitative data may ‘de-contextualise findings and wrongly assume that these are commensurable’ [25]. Nonetheless, by combining this disparate data, the identified themes have been developed from a broader body of research than any single study, and may therefore increase reliability of the results [24].

### Implications for policy and practice

As demonstrated by this review, health-seeking behaviour for schistosomiasis is affected by a number of factors. Health education programmes designed to improve such behaviour must recognise that increasing an individual's biomedical knowledge alone is unlikely to change their behaviour [12]. Strategies need to acknowledge and be sensitive to local perceptions of schistosomiasis, particularly in relation to perceived severity and, if necessary, tackle the possible stigma surrounding the infection.

Health education programmes that adapt to the local conditions of the area are more likely to receive greater attention and have a higher chance of impact on behaviour [14]. Nonetheless, if people do not have the means for changing behaviour (e.g. inadequate health services, unavailability of anti-helminth medications, and unaffordability of modern treatment), health education will only have a limited impact on health-seeking behaviour.

### Implications for future research

Many of the articles included within this review were predominantly descriptive: health-seeking behaviour in relation to schistosomiasis is detailed, while attempts at identifying the factors that underlay the behaviour are limited. Future research needs to move beyond documenting health-seeking behaviour, to understanding how factors influencing such behaviour are formed and changed. Moreover, given that this review has identified the local environment and community as a determinant for seeking treatment, it is pertinent to explore the social context in which these behaviours occur.

Further study should also investigate if stigma surrounding the infection is an issue in other communities where schistosomiasis is present. Additionally, in areas that have previously undergone external schistosomiasis control measures, the health-seeking behaviour of communities should be examined.

Limited information from the included studies was supplied with respect to age, gender, and locus of decision making in relation to the use and non-use of health services for schistosomiasis. Future research should attempt to address these gaps, and explore how and why these factors may influence whether treatment is obtained or not.

## Conclusion

Passive case finding at modern health facilities for schistosomiasis represents an important aspect of its control. Through analysing research of varying methodologies, this review has identified a range of factors that influence use and non-use of modern health facilities for the infection. In particular, it has highlighted potential stigma surrounding the infection, and aspects of the local environment and community, both of which may result in health services not being utilised. Health education programmes that intend to improve health-seeking behaviour for schistosomiasis need to acknowledge the multiple determinants influencing such behaviour. Future research needs to move beyond documenting health-seeking behaviour into examining the reasons behind such behaviour, as well as addressing a number of the gaps that have been highlighted from the review.

## Additional material

**Annex 1:** Characteristics and assessments of the quality for the included studies

## References

[1] Jamison DT (2006). Disease Control Priorities in Developing Countries.

[2] Hotez PJ, Kamath A (2009). Neglected tropical diseases in sub-saharan Africa: review of their prevalence, distribution, and disease burden. PLoS neglected tropical diseases.

[3] Steinmann P, Keiser J, Bos R, Tanner M, Utzinger J (2006). Schistosomiasis and water resources development: systematic review, meta-analysis, and estimates of people at risk. The Lancet infectious diseases..

[4] King CH, Dangerfield-Cha M (2008). The unacknowledged impact of chronic schistosomiasis. Chronic illness..

[5] Hotez PJ, Fenwick A (2009). Schistosomiasis in Africa: an emerging tragedy in our new global health decade. PLoS neglected tropical diseases..

[6] WHO Diseases covered by NTD department. http://www.who.int/neglected_diseases/diseases/en/..

[7] The control of schistosomiasis (1993). Second report of the WHO Expert Committee. World Health Organization technical report series.

[8] WHO (2009). The social context of schistosomiasis and its control: an introduction and annotated bibliography. http://whqlibdoc.who.int/publications/2008/9789241597180_eng.pdf..

[9] Engels D, Chitsulo L, Montresor A, Savioli L (2002). The global epidemiological situation of schistosomiasis and new approaches to control and research. Acta Trop..

[10] Committee WHOE (2002). Prevention and control of schistosomiasis and soil-transmitted helminthiasis. World Health Organization technical report series.

[11] Guyatt HL, Evans D (1992). Economic considerations for helminth control. Parasitology today..

[12] Mackian S, Bedri N, Lovel H (2004). Up the garden path and over the edge: where might health-seeking behaviour take us?. Health policy and planning..

[13] Shaikh BT (2008). Understanding social determinants of health seeking behaviours, providing a rational framework for health policy and systems development. JPMA The Journal of the Pakistan Medical Association..

[14] Aagaard-Hansen J, Mwanga JR, Bruun B (2009). Social science perspectives on schistosomiasis control in Africa: past trends and future directions. Parasitology..

[15] Kloos H (1995). Human behavior, health education and schistosomiasis control: a review. Social science & medicine..

[16] Huang YX, Manderson L (2005). The social and economic context and determinants of schistosomiasis japonica. Acta tropica..

[17] Mulrow CD (1994). Rationale for systematic reviews. Bmj..

[18] Centre for Reviews and Dissemination. University of York (2009). Undertaking Systematic Reviews of Research on Effectiveness: CRD's Guidance for those Carrying Out or Commissioning Reviews. http://www.york.ac.uk/inst/crd/pdf/Systematic_Reviews.pdf.

[19] Dixon-Woods M, Sutton A, Shaw R, Miller T, Smith J, Young B (2007). Appraising qualitative research for inclusion in systematic reviews: a quantitative and qualitative comparison of three methods. Journal of health services research & policy..

[20] Daly J, Willis K, Small R, Green J, Welch N, Kealy M (2007). A hierarchy of evidence for assessing qualitative health research. Journal of clinical epidemiology..

[21] Barbour RS (2001). Checklists for improving rigour in qualitative research: a case of the tail wagging the dog?. Bmj..

[22] Hawker S, Payne S, Kerr C, Hardey M, Powell J (2002). Appraising the evidence: reviewing disparate data systematically. Qualitative health research..

[23] Dixon-Woods M, Agarwal S, Jones D, Young B, Sutton A (2005). Synthesising qualitative and quantitative evidence: a review of possible methods. Journal of health services research & policy..

[24] Lucas PJ, Baird J, Arai L, Law C, Roberts HM (2007). Worked examples of alternative methods for the synthesis of qualitative and quantitative research in systematic reviews. BMC medical research methodology..

[25] Thomas J, Harden A (2008). Methods for the thematic synthesis of qualitative research in systematic reviews. BMC medical research methodology..

[26] el Katsha S, Watts S (1997). Schistosomiasis in two Nile delta villages: an anthropological perspective. Tropical medicine & international health: TM & IH..

[27] Aryeetey ME, Aholu C, Wagatsuma Y, Bentil G, Nkrumah FK, Kojima S (1999). Health education and community participation in the control of urinary schistosomiasis in Ghana. East African medical journal..

[28] Anguzu J, Oryema-Lalobo M, Oundo GB, Nuwaha F (2007). Community perception of intestinal schistosomiasis in Busia district of Uganda. East African medical journal..

[29] Acka CA, Raso G, N'Goran EK, Tschannen AB, Bogoch II, Seraphin E (2010). Parasitic worms: knowledge, attitudes, and practices in Western Cote d'Ivoire with implications for integrated control. PLoS neglected tropical diseases..

[30] Mwanga JR, Magnussen P, Mugashe CL, Gabone RM, Aagaard-Hansen J (2004). Schistosomiasis-related perceptions, attitudes and treatment-seeking practices in Magu district, Tanzania: public health implications. Journal of biosocial science.

[31] Mwanga JR (2005). Perceptions and practices on schistosomiasis among communities in Ukerewe district, Tanzania. Tanzan Health Research Bulletin.

[32] Yirenya-Tawiah DR, Annang T, Otchere J (2011). Urinary schistosomiasis among adults in the Volta Basin of Ghana: prevalence, knowledge and practices. Journal of Tropical Medicine and Parasitology..

[33] Ahlberg BM MR, Poggensee G, Feldmeier H, Krantz I (2003). “Better infection than hunger” A study of illness perception with special focus on urinary schistosomiasis in Northern Tanzania. African Sociological Review.

[34] Onyeneho NYP, Egwuage J, Emukah E (2010). Perceptions, attitudes and practices on schistosomiasis in Delta State, Nigeria. Tanzania Journal of Health Research..

[35] Hewlett BS, Cline BL (1997). Anthropological contributions to a community-based schistosomiasis control project in northern Cameroun. Tropical medicine & international health: TM & IH..

[36] Danso-Appiah A, De Vlas SJ, Bosompem KM, Habbema JD (2004). Determinants of health-seeking behaviour for schistosomiasis-related symptoms in the context of integrating schistosomiasis control within the regular health services in Ghana. Tropical medicine & international health: TM & IH.

[37] Gazzinelli A, Gazzinelli MF, Cadete MM, Pena Filho S, Sa IR, Kloos H (1998). Sociocultural aspects of schistosomiasis mansoni in an endemic area in Minas Gerais, Brazil. Cadernos de saude publica..

[38] Adeneye AK, Akinwale OP, Idowu ET, Adewale B, Manafa OU, Sulyman MA (2007). Sociocultural aspects of mass delivery of praziquantel in schistosomiasis control: the Abeokuta experience. Research in social & administrative pharmacy: RSAP..

[39] Kloos H, Ouma JH, Kariuki HC, Butterworth AE (1987). Coping with intestinal illness among the Kamba in Machakos, Kenya, and aspects of schistosomiasis control. Social science & medicine..

[40] Akinwale OP AA, Sulyman MA, Idowu ET, Adewale B (2005). Health care seeking behaviour of people in schistosomiasis endemic communities of Ogun and Niger State, Nigeria. Nigerian Journal of Parasitology..

[41] Uchoa E, Barreto SM, Firmo JO, Guerra HL (2000). The control of schistosomiasis in Brazil: an ethnoepidemiological study of the effectiveness of a community mobilization program for health education. Social science & medicine.

[42] Danso-Appiah A, Stolk WA, Bosompem KM, Otchere J, Looman CW, Habbema JD (2010). Health seeking behaviour and utilization of health facilities for schistosomiasis-related symptoms in ghana. PLoS neglected tropical diseases..

[43] Takougang I, Meli J, Fotso S, Angwafo F, Kamajeu R, Ndumbe PM (2004). Some social determinants of urinary schistosomiasis in Northern Cameroon: implications for schistosomiasis control. African journal of health sciences.

[44] Reis DC, Kloos H, King C, Quites HF, Matoso LF, Coelho KR (2010). Accessibility to and utilisation of schistosomiasis-related health services in a rural area of state of Minas Gerais, Brazil. Memorias do Instituto Oswaldo Cruz..

[45] Kroeger A (1983). Anthropological and socio-medical health care research in developing countries. Social science & medicine..

[46] Shaikh BT, Hatcher J (2005). Health seeking behaviour and health service utilization in Pakistan: challenging the policy makers. Journal of public health..

[47] McCombie SC (1996). Treatment seeking for malaria: a review of recent research. Social science & medicine.

[48] Williams HA, Jones CO (2004). A critical review of behavioral issues related to malaria control in sub-Saharan Africa: what contributions have social scientists made?. Social science & medicine..

[49] WHO (2002). Traditional Medicine Strategy 2002-2005. http://whqlibdoc.who.int/hq/2002/who_edm_trm_2002.1.pdf.

